# A comprehensive congenital syphilis case report with evidence-based insights into current practices

**DOI:** 10.25122/jml-2024-0363

**Published:** 2025-04

**Authors:** Ramona Mohora, Alexandra Diaconu, Silvia-Maria Stoicescu, Octaviana Cristea

**Affiliations:** 1 Department of Neonatology, Alessandrescu-Rusescu National Institute for Mother and Child Health, Polizu Maternity, Bucharest, Romania; 2 Carol Davila University of Medicine and Pharmacy, Bucharest, Romania; 3 Neonatal Transport Unit, Alessandrescu-Rusescu National Institute for Mother and Child Health, Polizu Maternity, Bucharest, Romania

**Keywords:** congenital infection, congenital syphilis, case report

## Abstract

Congenital syphilis is one of the most well-known congenital infections. Despite notable progress in early diagnosis of syphilis paired with the accessibility of cost-effective treatment and preventive strategies, a few cases continue to be diagnosed in the department of obstetrics. This paper presents a case study of an infant with low birth weight, delivered by an adolescent mother, part of a marginalized demographic group. Due to the mother's lack of routine prenatal care, the infant's management required a series of investigations to establish a comprehensive differential diagnosis. Maternal serological assessments for syphilis, including both non-treponemal antibody test (RPR) and treponemal antibody test (TPHA), yielded positive results following fetal extraction via cesarean section, specifically after diagnosis of syphilis in the infant. Within the first 24 hours of life, newborn serologic tests for syphilis (STS) (including RPR and TPHA assays) exhibited reactivity with titers equivalent to maternal samples. Furthermore, at three weeks of life, the neonatal STS titer exceeded that of the maternal titer, displaying a fourfold increase over the maternal STS level. This finding was concomitant with the detection of IgM antibodies against T. pallidum. Screening for other congenital infections yielded negative results. Subsequent to the high-risk infant follow-up, in accordance with the National Guidelines, the infant had a good outcome.

## INTRODUCTION

Syphilis, a sexually transmitted infection caused by *Treponema pallidum (T. pallidum)*, can be transmitted from mother to child during pregnancy, leading to congenital syphilis (CS) [1]. The number of CS cases in Europe has been decreasing since the early 2000s; however, in 2018 there was a notable increase in reported CS cases. In Romania, the most recent reported cases occurred in 2018, with four cases documented, with a rate of 1.9 per 100,000 live births [2].

Vertical transmission from an infected mother to her child typically occurs prenatally, often involving placental passage. However, perinatal transmission (via contact with infectious lesions in the birth canal or perineum) and postnatal transmission (although rare) have also been described. The extent of fetal damage is presumed to depend on the stage of fetal development at the time of infection and the time elapsed before treatment initiation [3]. The risk of fetal transmission is strongly correlated with the timing of maternal infection; when syphilis is acquired early in pregnancy, the risk of transmission is significantly higher—a principle known as Kassowitz’s law [3,4]. The risk remains elevated if maternal treatment is administered within four weeks of delivery. During the primary and secondary stages of syphilis, the likelihood of vertical transmission from an untreated woman is exceptionally high, approaching 100%. This risk decreases as the disease progresses, falling to approximately 10%–30% during the late latent stage [4].

In early CS, newborns may present as small for gestational age. Clinical features of CS often mirror those seen in other congenital infections, which imply the hematological spreading of the microorganism. The clinical manifestations of early CS typically emerge within the first 2 to 8 weeks of life and result from inflammation induced by the active infection [3]. Similar to the acquired form of syphilis, CS involves an intense inflammatory response primarily affecting the perivascular environment rather than the parenchyma [3]. Histologically, the umbilical cord may exhibit necrotizing funisitis, characterized by significant inflammation and perivascular bands of necrosis in Wharton jelly. Macroscopically, the umbilical cord may display edematous regions with spiral-striped red and pale blue patterns interposed with chalky white streaks resembling a 'barber's pole' [3].

At this stage, dermatological findings often include a maculopapular skin rash followed by desquamation, blistering, and crusting, prominently observed on the palms and soles. Infants affected by CS may have signs and symptoms of acute meningitis (e.g., bulging fontanel, vomiting), as well as 'salt and pepper' chorioretinitis [5]. Other complications may include pneumonia alba (obliterative fibrosis) [5,6], pulmonary hemorrhage [6], respiratory distress [6,7], myocarditis, hepatosplenomegaly resulting from extramedullary hematopoiesis [3,5], hepatitis [6], jaundice [6-8], ascites [3,8], necrotizing enterocolitis [3], fever, and pallor [6]. Approximately 2 to 3 months after infection, some infants may develop signs suggestive of immune complex-mediated nephrotic syndrome [5]. Symptomatic infants may also present with sepsis caused by other bacteria, including *Escherichia coli, Yersinia* species, and group B streptococci – it is presumed to be secondary to the breakdown of the gastrointestinal mucosal barrier [3].

Hematological abnormalities commonly include anemia (both hemolytic and nonhemolytic), leukopenia, leukocytosis, thrombocytopenia [5], hypoalbuminemia, and hypoproteinemia [1]. Cerebrospinal fluid analysis (CSF) may reveal pleocytosis, elevated protein levels, low glucose levels, and a reactive venereal disease research laboratory (VDRL) test. Bone involvement may manifest with symmetrical long bone lesions, more frequently observed in the lower limbs, along with Wimberger’s sign (demineralization and destruction of the proximal tibial metaphyses) [5].

Some clinical manifestations of late CS are interstitial keratitis, secondary glaucoma [3], sensorineural hearing loss due to osteochondritis of the otic capsule and cochlear degeneration, frontal bossing, square cranium (skull bone periostitis) [5] and mental retardation [3]. Early treatment of CS has almost eliminated the incidence of these cases in developed countries; however, late CS still occurs in approximately 40% of untreated survivors [3].

It is estimated that more than half of all infected neonates are asymptomatic at birth. Therefore, maternal testing and treatment history are crucial, in addition to clinical and laboratory findings [7]. Most newborns acquire non-treponemal antibodies (VDRL/RPR) transplacentally, which typically disappear by 12 to 18 months [5]. A reactive non-treponemal test in infants with classic symptoms of CS suggests a presumptive diagnosis; however, to exclude a false-positive test result, a positive non-treponemal test should be confirmed by a specific treponemal test [4]. A rapid plasma reagin (RPR) titer in the newborn fourfold higher than the maternal titer strongly suggests CS; nevertheless, lower titers do not definitively exclude the diagnosis (Table 1) [9,10].

**Table 1:** Maternal and infant serologic test results [10]

	Treponemal (TP-PA)		Non-treponemal (VDRL, RPR)		
	Infant	Mother	Infant		Mother
Situation 1	+	+	+	-	+
Situation 2	-	-	+		
Situation 3	+	+	-		-

Situation 1 = mother with recent/precious syphilis/latent infection AND possible syphilis in infant

Situation 2 = false positive non-treponemal test, no syphilis infection in mother or infant

Situation 3 = mother with syphilis successfully treated in early pregnancy or before / false-positive serologic tests (e.g., Pinta, Yaws, Lyme disease)

More than 80% of symptomatic infants have immunoglobulin M (IgM) antibodies. Nevertheless, serological results must be interpreted cautiously, as IgM responses may take time to develop in infants and can be reduced with early treatment. Therefore, a negative IgM result should not be used to exclude congenital infection [9]. Symptomatic infants with negative IgM should undergo retesting at 4 and 8 weeks, as there can be a delayed IgM response [11].

In infants presenting with symptoms or born to mothers with untreated syphilis, diagnosis can be confirmed through direct detection of *T. pallidum* using microscopy or molecular techniques. The organism may be identified in specimens obtained from lesions, ulcers, nasopharyngeal secretions, CSF, or other body tissues. However, due to the limited sensitivity of microscopy techniques, a positive microscopy finding confirms the diagnosis of CS, while a negative one does not rule out the disease [9]. CSF analysis is valuable for assessing possible neurosyphilis. Testing should include CSF VDRL, total protein concentration, and white blood cell count. Interpretation of VDRL results must be approached with caution, as a negative CSF VDRL does not definitively rule out neurosyphilis [3].

## CASE PRESENTATION

In this paper, we aim to present a case of CS, focusing on its signs despite the variability of its manifestations, and emphasize the low incidence in our country [2]. Adequate prenatal care is crucial, especially in promptly identifying and managing newborns at risk for late CS [3]. At the same time, we would like to raise awareness that infants born from pregnancies lacking prenatal care incur higher costs due to increased requirements for case management. Furthermore, we would like to highlight the issue of inadequate prenatal care among low-income patients, particularly adolescent patients, which can result in negative fetal health outcomes.

In 2018, 18,753 adolescent girls aged 15–19 years in Romania became mothers, of whom 3,929 were giving birth to their second child. Additionally, 727 girls under 15 became mothers, 19 of whom were second-time mothers. In 2019, the total number of adolescent pregnancies decreased. However, adolescent mothers continue to experience health issues during pregnancy more frequently than adult mothers. These health issues include anemia, pregnancy-induced hypertension, and intrauterine growth restriction. Romania ranks among the top two countries in the European Union with the highest adolescent birth rates, second only to Bulgaria [12].

We present the case of a male infant born to a 16-year-old mother, gravida 4, with a history of spontaneous abortion. The mother was admitted in active labor to the Alessandrescu-Rusescu National Institute for Mother and Child Health, Polizu Maternity. Despite residing in an urban area with the child’s father, she had not sought any prenatal medical care during the pregnancy. Upon admission, medical examination revealed oligohydramnios and meconium-stained amniotic fluid. Due to signs of acute fetal distress, a cesarean section was performed. A male newborn was delivered, weighing 2,330 grams, with a gestational Ballard score corresponding to 42–43 weeks. The Apgar score was 8 at 1 minute and 7 at 5 minutes. The newborn was classified as small for gestational age and presented with disharmonious physical development.

**Figure 1:**
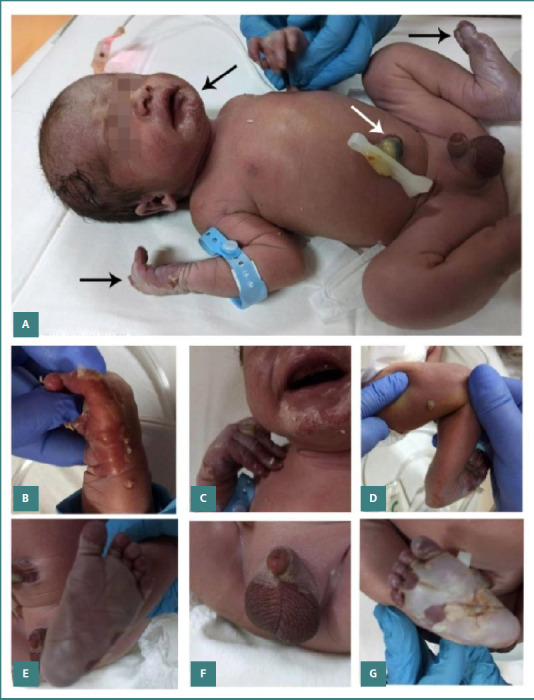
Physical examination findings at birth in a neonate with extensive skin involvement. A, Extended wet desquamation (black arrows) and green-stained umbilical cord (white arrows); B and C, Upper limb desquamation (palmar region) and perioral lesions with bullous aspect and desquamation; D, Unbroken skin over a blister (fluid-filled lesion) on the thigh; E, Desquamation of the soles, more severe on the right side; F, Genital involvement, G, Lower limbs with extensive desquamation on the sole region.

The initial clinical examination revealed moist skin desquamation (pemphigus syphiliticus) localized on the palms, soles, scrotum, and face, and blistering on the right thigh and the third finger of both upper and lower limbs (Figure 1 A-G). Over time, the moist desquamation evolved into dry desquamation, with ichthyosiform changes in the skin. In addition to the dermatological findings, palpable hepatosplenomegaly was observed. The newborn displayed suboptimal adaptation from intrauterine to extrauterine life, necessitating admission to the Neonatal Intensive Care Unit (NICU) due to respiratory distress. Patient management included the initiation of non-invasive respiratory support with a Heated Humidified High-Flow Nasal Cannula (HHHFNC) for one day, followed by supplementation with free-flow oxygen (30%) for three days. During the NICU stay, hyperchromic urine was observed, which subsequently normalized prior to discharge. In the first hours of life, laboratory investigations revealed severe thrombocytopenia (34.00 x 10^3^/ul), platelet aggregates observed on blood smear examination, and mild anemia (Table 2). Serological tests indicated a 1/16 RPR titer and reactive TPHA (Table 3). Initially, the newborn’s syphilis serology titer mirrored that of the maternal titer; however, subsequent serological testing of the newborn indicated a fourfold increase compared to the mother's titer. Following the completion of treatment, the RPR titer decreased. Analysis of CSF indicated non-reactive RPR and TPHA, elevated protein levels, and a clear and colorless appearance with a negative Pandy test and a negative culture result. Serological tests for HIV, rubella, cytomegalovirus, and toxoplasmosis were negative. Additionally, no pathological microorganisms were detected in newborn blood and skin cultures or samples collected from the mother’s uterine cavity and cervical secretions.

**Table 2:** Longitudinal profile of paraclinical laboratory findings from birth to 3.5 months of life

Parameter	24 hours	Day 7	Week 2	Month 1	1.5 Months	Month 2	Month 3	3.5 Months
Hemoglobin (g/dL)	11.60 ↓	14.70	15.20	9.20 ↓	9.70 ↓		10.80	
Hematocrit (%)	40.80	48.20	48.50	29.70 ↓	29.70 ↓		32.00	
Thrombocytes (x 10^3^/ul)	34.00 ↓	89.00 ↓	305.00	458.00	437.00		467.00 ↑	
α1-Fetoprotein (IU/ml)				19981 ↑				
AST (u/L)	133.00	53.00	260.00 ↑	213.00 ↑	288.00 ↑	112.80 ↑	70.00	49.90
ALT(u/L)	28.00	22.00	218.00 ↑	221.00 ↑	357.00 ↑	118.70 ↑	112.00 ↑	68.60 ↑
GGT (u/L)	250.00 ↑	157.00	111.00	180.00			75.00	
LDH (u/L)	2404.00 ↑			317				
C-Reactive protein (mg/dL)	6.10 ↑	1.0	0.8	0.0			0.00	
Procalcitonin		< 0.5	<0.5	<0.5				
Total Bilirubin (mg/dL)	4.49 ↑	7.95 ↑	8.18 ↑	6.66 ↑	5.70 ↑	4.55 ↑	0.8	0.78
Indirect Bilirubin (mg/dL)	3.06 ↑	3.24 ↑	2.85 ↑	1.50 ↑	1.1 ↑	1.18 ↑	0.3	0.32
Direct Bilirubin (mg/dL)	1.43 ↑	4.71 ↑	5.33 ↑	5.16 ↑	4.60 ↑	3.37 ↑	0.5 ↑	0.46 ↑

AST, aspartate aminotransferase; ALT, alanine aminotransferase; GGT, gamma-glutamyl transferase; LDH, lactate dehydrogenase.

↑ = elevated level; ↓ = decreased level; (missing symbol = normal range).

**Table 3:** Longitudinal serologic titers in mother and infant during the first 3.5 months of life

Time points	TPHA		RPR (VDRL carbon)	
	Newborn	Mother	Newborn	Mother
24 hours	1/80	1/80	1/16	1/16
3 weeks	1/80		1/64	
1 month	1/80		1/32	
1 month + 1 week	1/80		1/16	
1.5 months	1/80		1/16	
1 months + 1 week (+)	1/640	1/640	1/64 (*)	1/8 (*)
3.5 months (+)	1/640	1/640	negative (*)	1/8 (*)

(*) = VDRL cantitative, (+) = different laboratory techniques

During hospitalization, laboratory findings included abnormal hepatic enzymes, positive inflammatory tests, and neonatal anemia. Immunoglobulin profiling revealed low IgG (675.00mg/dL), elevated IgM (77.00 mg/dL), and normal IgA (27.90 mg/dL). These changes normalized during hospitalization, although some quasi-constant elevated levels of direct bilirubin and hepatic enzymes persisted.

**Figure 2:**
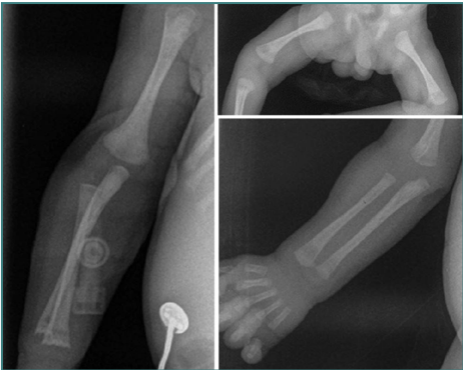
Radiographic findings of the long bones in a neonate

After birth, a radiological examination revealed several findings in the long bones: lucent metaphyseal bands of the proximal and distal radius and ulna, along with slight irregularities in the metaphysis (Figure 2). The 'saw tooth' appearance of metaphyseal dystrophy is a specific sign observed in CS [13].

**Figure 3:**
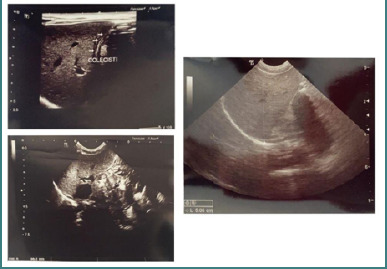
Abdominal ultrasound at 1 month of life. The liver demonstrates homogeneous echotexture, smooth surface contour, and normal size. The spleen appears enlarged with coarse echotexture, consistent with persistent clinical splenomegaly.

The cranial ultrasound investigation detected bilateral abnormal areas near the lateral ventricles, suggesting intrauterine grade I intraventricular hemorrhage (IVH), specifically subependymal/germinal matrix hemorrhage (SHE/GMH). Initially, an abdominal ultrasound investigation identified small hyperechoic lesions in the spleen (Figure 3). Serial ultrasounds were performed until discharge, and the lesions were no longer evident. Additionally, ophthalmological and audiological assessments were normal.

**Figure 4:**
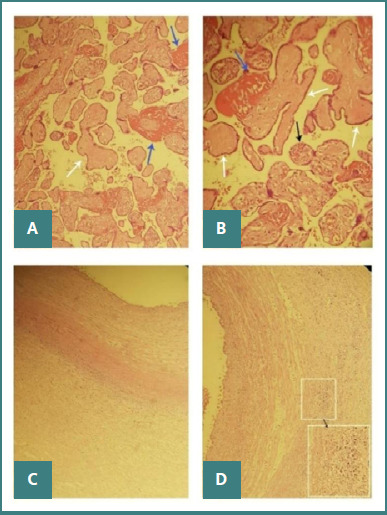
Histopathological examination of the placenta and umbilical cord in congenital syphilis. A and B, Sections of chorionic villi demonstrating avascular villi (white arrows), fibrin deposition (blue arrows), and an increased number of capillaries (black arrows); C and D, Sections of the umbilical cord showing moderate edema and inflammatory cell infiltrate, consistent with funisitis.

The histopathological examination of the placenta and umbilical cord revealed specific features (Figure 4A-D). The fetal face of the placenta displayed congested blood vessels along with small-sized chorionic villi, some of which were poorly vascularized. Several areas exhibited avascular chorionic villi with sclerosis and hyalinization, interspersed with regions of increased vascularization (approximately 10 capillaries per chorionic villus). The subchorionic placenta exhibited moderate fibrinous exudate and inflammatory infiltrate, primarily consisting of polymorphonuclear and neutrophils. The umbilical cord displayed moderate edema localized within Wharton's jelly and significant inflammation, suggesting funisitis. Additionally, focal microcalcifications and lesions of subchorionitis were observed.

After admission to the NICU, the newborn required noninvasive respiratory support, total parenteral nutrition (TPN), and enteral nutrition starting from the second day of life. As per national guidelines, specific antibiotic therapy for suspected CS was initiated immediately.

**Figure 5:**
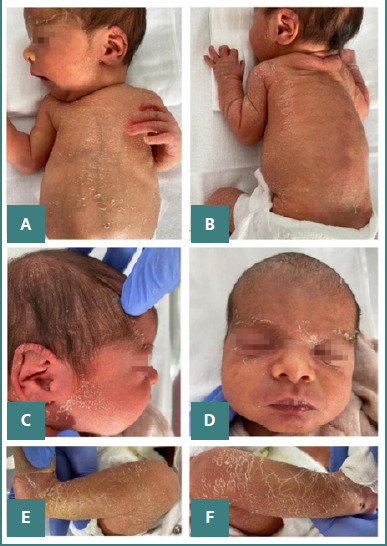
Physical examination findings during hospitalization. A and B, Ichthyosiform eruption involving the trunk and back, with abdominal distension and palpable hepatosplenomegaly; C and D, Scaling of the face with relative sparing of the zygomatic, nasal, and perioral regions; E and F, Severe ichthyosiform involvement of the lower limbs.

**Figure 6:**
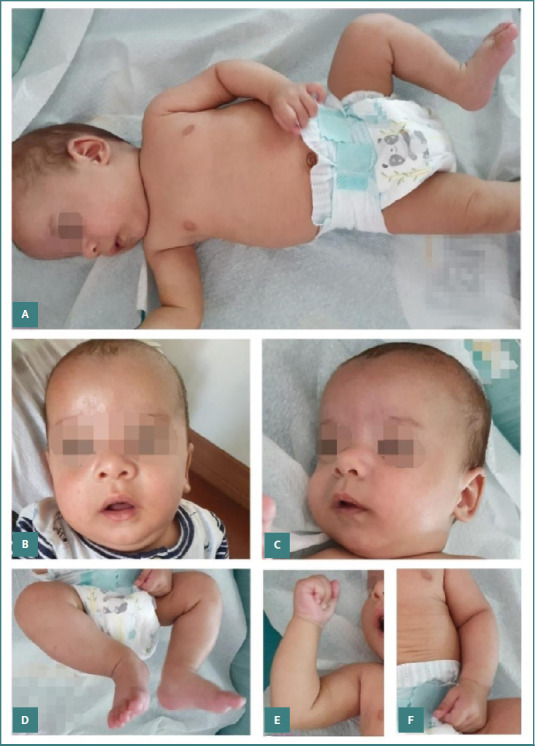
Physical examination at 3-month follow-up after hospital discharge. Normal growth and neurodevelopment were observed. The skin shows complete epithelialization and a healthy appearance across all regions: A, Full-body view with normal skin integrity; B and C, Face with well-healed, healthy skin; D, Lower limbs with normal texture and tone; E and F, Upper limbs with no residual lesions or abnormalities.

The outcome was good, with improvement noted in both laboratory parameters and the newborn's general condition. Treatment and specialized care continued after NICU discharge, and the newborn was successfully weaned off oxygen and transitioned to exclusive enteral nutrition. Dermatological manifestations evolved into dry, scaly skin with desquamation (Figure 5 A-F). Gradually, the patient completely recovered and was discharged after a total of 47 days since admission. Long-term multidisciplinary follow-up was provided (Figure 6 A-F).

## DISCUSSION

This case report highlights the diverse clinical manifestations, complex neonatal diagnosis, and extensive follow-up required for newborns with CS. The patient exhibited well-recognized typical clinical, laboratory, and radiographic features of CS. The nontreponemal titer was fourfold higher than the mother’s, with positive IgM *Treponema pallidum* (Blot) testing.

Other conditions presenting with hepatosplenomegaly and cholestatic jaundice were considered, including viral hepatitis (ruled out via negative serology), isoimmunization (excluded due to no blood group and Rh incompatibility), biliary tract obstruction (no suggestive sonographic findings) and endocrine disorders (hypothyroidism excluded by Guthrie test and hormonal levels assessment) [14]. Additionally, hereditary pathologies associated with vesiculobullous eruptions (such as bullous epidermolysis, Langerhans histiocytosis, acrodermatitis enteropathica) [3] and other causes of ichthyosis were considered as a possible diagnosis. The comprehensive differential diagnosis reaffirms the reputation of CS as “the great imitator” in clinical practice [3]. The incidence of syphilis has been steadily rising over the past years [2]. CS often presents clinical similarities with other diseases. Given the absence of prenatal medical care, the presence of meconium-stained amniotic fluid, poor neonatal transition, and severe thrombocytopenia with acute inflammation signs, neonatal sepsis was initially suspected. CS may also closely resemble other congenital infections, leading to the exclusion of toxoplasma, rubella, cytomegalovirus, hepatitis B, hepatitis C, and HIV infection. In Romania, syphilis screening is a standard part of routine pregnancy care [15], offering effective diagnosis and treatment to prevent maternal-fetal transmission, which is simpler than preventing HIV transmission. While the screening procedure is simple, implementing the screening program may pose challenges [16]. No newborn should be discharged from the hospital without documentation of maternal syphilis serology status. Positive infants and those born to positive mothers require clinical and serological follow-up every 2–3 months until the tests return negative results or titers decrease by a factor of four. A positive VDRL result in CSF or abnormal cell count and/or protein concentrations requires initiation of treatment for possible neurosyphilis [15] and ongoing pediatric neurology follow-up. The persistence of the pathology may be attributed to significant difficulties in accessing healthcare among disadvantaged groups [17].

## CONCLUSION

The rising incidence of syphilis today highlights the importance of recognizing CS. The multidisciplinary management required for diagnosis and follow-up poses significant challenges for clinicians. Despite the cost-effectiveness of antenatal screening, many pregnant women do not benefit from it due to various factors such as educational, social, and financial constraints. CS and other congenital infections should be considered in cases where prenatal records are unavailable. Clinicians may have limited familiarity with the diagnosis and management of this disease. Furthermore, the broad spectrum of clinical manifestations in CS infection mimics many other conditions, increasing the risk of misdiagnosis. Early diagnosis of CS is crucial to reducing the incidence of stillbirth, premature labor, and perinatal mortality.

According to the Romanian National Center for Surveillance and Control of Communicable Diseases, a healthcare committee comprising various medical specialties (including neonatology, dermatology, and epidemiology) must confirm all cases of CS. We emphasize that this case report contributes to the limited literature on CS, particularly considering the current global low incidence rates. In conclusion, reducing the incidence of neonatal and infant mortality and morbidity requires comprehensive support for optimal prenatal care and pediatric follow-up programs.

## Personal thanks

The authors would like to gratefully acknowledge Raluca Chirculescu, M.D., for examining histopathological samples and generating scanned images used in this paper, and Roxana Cebuc, M.D., Florin Brezan, M.D., and Alina Suru, M.D., for interdisciplinary collaboration.
